# Multiple Degrees of Freedom in the Fish Skull and Their Relation to Hydraulic Transport of Prey in Channel Catfish

**DOI:** 10.1093/iob/obaa031

**Published:** 2020-11-10

**Authors:** A M Olsen, L P Hernandez, E L Brainerd

**Affiliations:** Department of Ecology and Evolutionary Biology, Brown University, 171 Meeting St, Box G-B 204, Providence, RI 02912, USA; Department of Biological Sciences, Science and Engineering Hall, The George Washington University, 800 22nd Street NW, Suite 6000, Washington, DC 20052, USA; Department of Ecology and Evolutionary Biology, Brown University, 171 Meeting St, Box G-B 204, Providence, RI 02912, USA

## Abstract

Fish perform many complex manipulation behaviors without hands or flexible muscular tongues, instead relying on more than 20 movable skeletal elements in their highly kinetic skulls. How fish use their skulls to accomplish these behaviors, however, remains unclear. Most previous mechanical models have represented the fish skull using one or more planar four-bar linkages, which have just a single degree of freedom (DoF). In contrast, truncated-cone hydrodynamic models have assumed up to five DoFs. In this study, we introduce and validate a 3D mechanical linkage model of a fish skull that incorporates the pectoral girdle and mandibular and hyoid arches. We validate this model using an *in vivo* motion dataset of suction feeding in channel catfish and then use this model to quantify the DoFs in the fish skull, to categorize the motion patterns of the cranial linkage during feeding, and to evaluate the association between these patterns and food motion. We find that the channel catfish skull functions as a 17-link, five-loop parallel mechanism. Despite having 19 potential DoFs, we find that seven DoFs are sufficient to describe most of the motion of the cranial linkage, consistent with the fish skull functioning as a multi-DoF, manipulation system. Channel catfish use this linkage to generate three different motion patterns (rostrocaudal wave, caudorostral wave, and compressive wave), each with its own associated food velocity profile. These results suggest that biomechanical manipulation systems must have a minimum number of DoFs to effectively control objects, whether in water or air.

## Introduction

Teleost fishes (subsequently referred to as “fishes”) can exert exquisite control over the flow of fluid and the motion of particles suspended in fluid into and within their mouths for behaviors such as filter feeding, mouthbrooding, suction feeding, and prey processing (Sibbing et al. 1986; Sanderson et al. 2001; Van Wassenbergh et al. 2016; Weller et al. 2017). Remarkably, fishes accomplish these complex manipulation tasks without hands or flexible muscular tongues (Dean et al. 2005), relying instead on the over 20 movable skeletal elements in their highly kinetic skulls including the mandibular and hyoid arches, the branchial apparatus that supports the gills, the shoulder girdle, and in some fishes the pharyngeal jaws. While some of these behaviors include direct manipulation of food particles through contact with the jaws and other skeletal elements, all of these behaviors depend to some extent on indirect manipulation achieved by controlling the unidirectional or bidirectional flow of fluid suspending these particles, termed “hydraulic transport” (Bemis and Lauder 1986).

Effective object manipulation depends on the ability to control all the degrees of freedom (DoFs) of an object (the total parameters needed to characterize its position and orientation). This implies that the mobility (total DoFs) of a motor system must be equal to or greater than the mobility requirement of the motor tasks that the system performs. This correspondence between task-required mobility and minimum internal mobility is apparent across robotic manipulation systems. For example, robotic arms that grasp, translate, and rotate objects in 3D space, a task requiring seven DoFs, have at least seven DoFs (Koch et al. 2018). And flow-based particle trapping systems that use fluid stream interactions to control the 2D translation of particles, a two-DoF task, have two valves (i.e., DoFs) for flow control (Tanyeri and Schroeder 2013). Based on this principle, fish skulls must have a minimum internal mobility of three DoFs to control 3D translation (changes in position along three axes) or six DoFs to control translation and rotation. This raises not only the question of how many DoFs a fish skull has but also the possibility that fish skulls may be a source of inspiration in improving the design of human-engineered flow-based manipulation systems. The mobility of the fish skull remains unresolved because while several models have been tested, these models have not assumed consistent mobilities. A classic approach has been to represent the skeletal elements, ligaments, and muscles of the skull as a series of connected rigid links or bars, most commonly as one or more planar, one-DoF four-bar linkages (Westneat 1990). In spite of many simplifying assumptions (that all links remain rigid, that all joints are one-DoF hinge joints, and that the entire system has just a single DoF), planar four-bar models have been found to accurately replicate *in vivo* motion, however only when measured against 2D motion data and for certain skeletal elements (Westneat 1990; Van Wassenbergh et al. 2005). For example, a 3D four-bar with at least three DoFs is needed to accurately represent the 3D *in vivo* motion of a linkage that depresses the mandible in largemouth bass (Olsen et al. 2017), suggesting that planar four-bar models underestimate the mobility of the fish skull.

In contrast to planar four-bar linkage models, hydrodynamic models that simulate the fluid flow patterns created by the skull suggest greater mobility. Expanding cone models (formed by one to three truncated cones) use two to four DoFs to control the rate of expansion along a hollow tube open at one end (Van Wassenbergh et al. 2006). And a computational fluid dynamics simulation that matches the prey velocities observed *in vivo* represents the head as a five-DoF deformable mesh (Van Wassenbergh 2015). The greater DoFs of these hydrodynamic models allow them to replicate the well-known rostrocaudal (RC) wave observed during suction feeding in fishes, a front-to-back sequential expansion that draws water into and through the mouth (Gibb and Ferry-Graham 2005; Bishop et al. 2008). Although the RC wave could be generated by passive coupling, our recent finding of a significant shift in the correlations among intracranial motions during suction feeding versus swallowing in channel catfish imply independent control, consistent with the fish skull acting as a high-DoF manipulation system (Olsen et al. 2019).

**Fig. 1:**
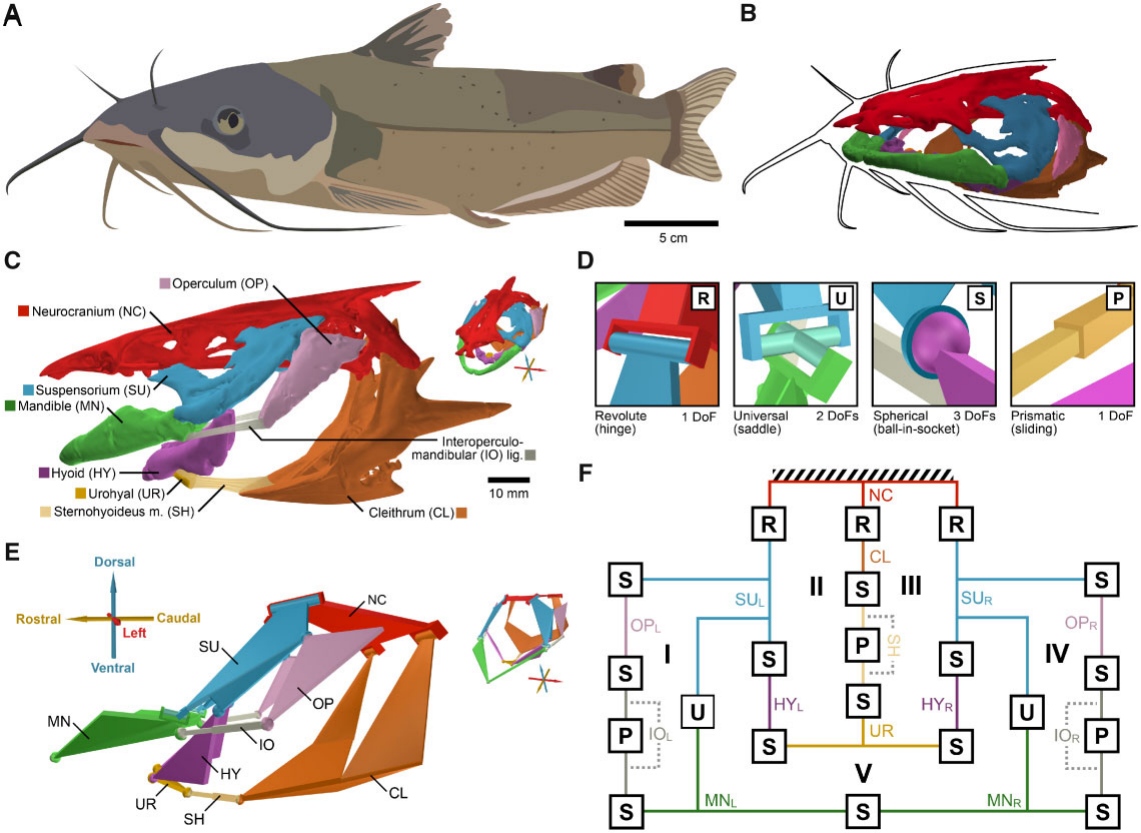
Anatomy of the channel catfish (*I. punctatus*) skull and its corresponding mechanism model. Unlike many other fishes, channel catfish have a relatively wide (dorsoventrally flattened) skull and mouth opening and long barbels that are used to detect food items (**A**). But similar to other fishes, channel catfish have kinetic skulls, with at least 11 mobile skeletal elements comprising the mandibular and hyoid arches and the shoulder (shown relative to the rostral body outline in (**B**) and labeled in (**C**)). Using four joint types (**D**), these elements can be represented as a five-loop parallel mechanism (shown as a linkage schematic in (**E**) and as a joint-and-loop graph in (**F**)). In (C) and (E), large renderings are shown in lateral view and smaller renderings in the upper right corner are shown in superolateral oblique view, with coordinate system arrows indicating anatomical axes and with corresponding colors and abbreviations. The four joint types (C) range from one to three DoFs (corresponding abbreviations in the upper right corner). In (F), lines correspond to links and boxes correspond to joints (angled, parallel lines indicate the fixed link, here, the neurocranium). Colors and link abbreviations in (F) correspond to those in (C) and (E), with subscripts “L” and “R” indicating left and right, respectively; roman numerals enumerate the five loops. Illustration in (A) by Aaron Olsen.

In this study, we propose a new 3D linkage model for the mandibular arch, hyoid arch, and shoulder girdle of fishes that combines the fidelity to anatomical structure and connectivity of mechanical linkage models with the capacity for serially independent motions of hydrodynamic models. We used 3D *in vivo* motion data collected during feeding in channel catfish (*Ictalurus punctatus*; Fig. 1A;  Olsen et al. 2019) to validate this model, to quantify the DoFs in the fish skull, and to test whether these cranial elements act as a prey manipulation system. If these elements function as a manipulation system then the *in vivo* motion of these elements should move with at least three DoFs for full translational control and at least six DoFs for full translational and rotational control. In addition, if these elements manipulate prey then motions of these elements should be associated with consistent patterns of prey motion.

## Materials and methods

### Animal care and surgical procedures

We used motion data that were collected and published in association with a previous study on motion integration (Olsen et al. 2019). For that study, we obtained channel catfish from Osage Catfisheries, Inc. (Osage Beach, MO, USA) and selected three individuals for motion data collection. These individuals (Indiv1, Indiv2, and Indiv3) had standard lengths (in cm) of 31.8, 30.5, and 37.5, respectively. After training the fish to feed on demand, we performed surgery to implant tantalum spherical markers for X-ray based motion tracking. We anesthetized the fish with buffered MS-222 (at 0.09–0.135 g/L) and administered an analgesic (0.4 mg/kg butorphanol). We unilaterally implanted 0.5 and 0.8 mm diameter markers into eight skeletal elements (Fig. 1B and C): neurocranium, urohyal, and left post-temporal, left cleithrum, left suspensorium, left operculum, left mandible, and left hyoid. Bone markers were implanted by pushing the markers into a hand-drilled hole having the same diameter as the markers. Animal care and procedures were approved by the Brown University Institutional Animal Care and Use Committee.

### *In vivo* data collection

We recorded synchronous X-ray videos during suction feeding from two views (biplanar fluoroscopy) at 300 frames per second. For filming, individuals were given three different prey types: half or whole live earthworms, dead squid pieces, and carnivore pellets; all prey were marked with a single tantalum marker to track prey motions throughout feeding. Our objective in presenting different prey types was to identify which prey type elicited the maximum intraoral pressure differential for a related study quantifying suction power (Camp et al. 2020). Thus we did not systematically present prey in such a way that would allow us to test for prey type effects. Trials collected from Indiv1 and Indiv2 used a mix of prey types (Indiv1: eight sinking pellets trials, three worm trials, and four squid trials; Indiv2: five sinking pellet trials, nine worm trials, one squid trial) whereas Indiv3 trials (12 total) used only worms since these were found to elicit the greatest pressure differential. Feeding behaviors recorded include prey capture, intraoral transport, and swallowing (transport into the esophagus).

### XROMM animation

To convert marker motions into 3D rigid-body transformations we used a workflow of marker tracking, reconstruction, and CT mesh unification known as X-ray Reconstruction of Moving Morphology (XROMM) animation (Brainerd et al. 2010), as described in a previous study (Olsen et al. 2019). Camera calibration, marker tracking, and marker reconstruction were performed using XMALab v1.3.9 (Knörlein et al. 2016). We segmented each skeletal element of interest from a CT scan (e.g., Fig. 1C) and exported marker coordinates in “CT space” using Horos v2.0.1 (horosproject.org). We performed all subsequent analyses using the R package “matools” (github.com/aaronolsen; R Core Team 2020). We smoothed X-ray marker trajectories and aligned (unified) the smoothed X-ray marker coordinates with their corresponding CT marker coordinates at each time frame to obtain a sequence of rigid-body transformations from CT to world space. The standard deviation in marker-to-marker distances within each skeletal element, a measure of precision, was 0.080 mm on average and mean unification errors did not exceed 0.15 mm for any skeletal element.

### Mechanism model construction

To construct our mechanism model, we first empirically determined the centers and axes of rotation between each pair of articulated skeletal elements by fitting joint models to their *in vivo* motion using the “fitMechanism” function in the R package “linkR” (Olsen and Westneat 2016), as described in a previous study (Olsen et al. 2019). We fit three joint models to each pair (Fig. 1D): a one-DoF hinge (revolute) joint, a two-DoF saddle (universal) joint, and a three-DoF ball-in-socket (spherical) joint. Model error was calculated as the root mean square (RMS) error between fit points (three landmarks distributed across each element) animated using the joint model versus fit points animated using *in vivo* rigid-body transformations. Each joint model was fit by iteratively optimizing the orientation and position of each axis, element pose, and rotations about each axis to minimize the model error. Since we had over 10,000 frames of motion data per individual we performed the optimization using 20 frames that represented the most disparate joint poses. We selected the lowest DoF model that surpassed certain error thresholds, as described in a previous study (Olsen et al. 2019).

We next combined the joint models for each articulated pair into a single mechanism model (Fig. 1E). Prismatic (sliding) joints with spherical joints at each end were added to represent compliant soft tissue elements: the left and right interoperculomandibular ligaments and the sternohyoideus muscle. The complete mechanism has a total of five loops (the number of unique, closed paths that can be “drawn” through connected links, indicated by roman numerals in Fig. 1F), 17 links (counting each prismatic pair as a link), and 21 joints, where the summed DoFs across all joints is 49. We calculated the total DoFs of the mechanism to be 19 using the Chebychev–Grübler–Kutzbach formula (Olsen 2019). Thus, 19 parameters are required to fully specify the conformation of the mechanism. Many combinations of 19 parameters are possible; we chose the parameterization that produced the simplest set of geometric constraint equations to simplify the computational simulations.

### Mechanism model fitting

**Fig. 2:**
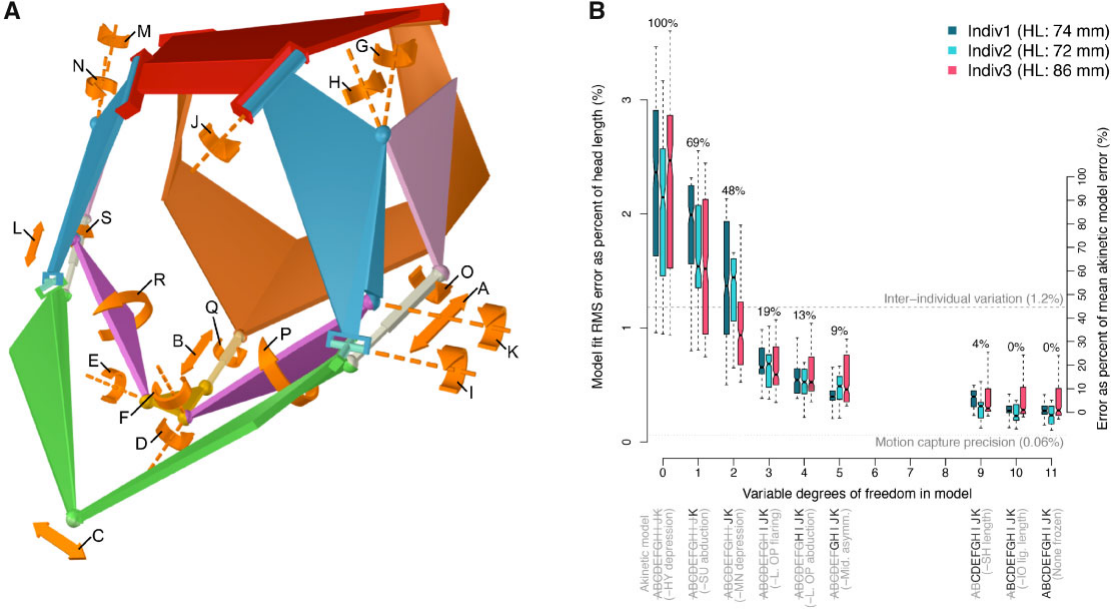
Mechanism model fit to *in vivo* motion. Although the channel catfish skull has at least 19 DoFs, just seven are sufficient to describe most of the motion during suction feeding. The channel catfish skull has 19 theoretical DoFs, indicated by orange arrows and letters A–S in (**A**). However, most of the motion during *in vivo* suction feeding behavior occurs along only a subset of these DoFs, as indicated by a non-linear relationship in (**B**) between model fit error (*y*-axis) and DoFs (*x*-axis). Fit errors are shown as box plots separated by individual fish, where the left *y*-axis is RMS error as a percent of head length (HL) and the right *y*-axis is error as a percent of the difference in error between the highest and lowest parameter models. Eight of the 19 DoFs were not measurable from our *in vivo* motion data (see “Materials and Methods” section) and thus we began fitting with an 11-DoF model, far right in (B), that allowed motion along the DoFs labeled AK. One or more DoFs were then successively frozen (from right to left along the *x*-axis), selecting the next DoF to freeze as that which increased fit error the least. The newly frozen DoF at each *x*-axis increment is noted in parentheses and all frozen DoFs are indicated by gray, strikethrough letters as labeled in (A). Each box-and-whisker summarizes 225 error measures (three fit points in five skeletal elements and 15 frames) with the percentage listed above indicating where the corresponding models fall on the right *y*-axis. The percentages for the two benchmarks (horizontal dashed and dotted lines) correspond to the left *y*-axis.

Although the mechanism has 19 DoFs, the magnitude of motion along these DoFs likely varies during feeding. We quantified the relative importance of each DoF by sequentially freezing each DoF in the mechanism (setting that DoF to its mean value) and fitting the resulting model to the 3D *in vivo* motion dataset. Of the 19 DoFs in the full-parameter mechanism model, eight were not measurable due to limitations in our motion capture dataset and thus frozen *a priori*. Of these eight DoFs, five DoFs represent long-axis rotations (or “twisting” for soft tissues) of links between two spherical joints (arrows labeled O–S in Fig. 2A); we did not have sufficient markers to quantify these motions. The remaining three DoFs that we could not measure represent right-side DoFs (arrows labeled L–N in Fig. 2A); since we only marked left-side elements we could not quantify these motions. Importantly, even though we only marked elements on the left side of the skull we could still assess whether motions were symmetric at the midline because the left mandible, the left hyoid, and the left cleithrum all extend to the midsagittal plane. Thus, we began the mechanism model fitting with an 11-DoF reduced parameter model.

The order in which to freeze the DoFs was determined by fitting models for each remaining unfrozen DoF and selecting the DoF that resulted in the smallest increase in error. In this way, we quantified model fit error as a function of variable DoFs in the model, with the DoFs ranked by their effect on model fit. The lowest parameter model (zero DoFs) represents akinesis (no intracranial motion). Each model was fit by iteratively optimizing the input DoFs to minimize the RMS error between model animated fit points and *in vivo* animated fit points. As with the joint model fitting, we had an excess of motion frames. Thus, for each individual, we performed the optimization using 15 frames representing the most disparate cranial conformations as we found that sampling greater than 15 frames did not consistently change the model fit error (Supplementary Fig. S4). The final RMS errors were scaled based on head length.

To assess how many DoFs are sufficient to capture the *in vivo* motion of this system (i.e., identify a best fitting mechanism model) we used three benchmarks. The first is a measure of precision of our motion capture data, calculated as the RMS difference between the mean marker configuration for each skeletal element and the marker configurations for that element in 50 random motion frames. This precision measure includes the effects of errors in 3D calibration, marker tracking, and non-rigidity of the skeletal elements. The second benchmark is a measure of inter-individual variation, calculated as the RMS difference in homologous landmark coordinates between each individual and a Procrustes-consensus shape. And the third benchmark is a percentile scale spanning the median error of the highest- and lowest-parameter models, which quantifies how much of the difference between the best and worst errors is explained by each model.

### Motion pattern analysis

Once we determined that five DoFs were sufficient to describe most of the motion in our unilateral *in vivo* dataset, we fit the five-DoF mechanism model to our entire *in vivo* motion dataset to characterize cranial linkage throughout all of our feeding trials. Importantly, these five DoFs do not represent simply joint rotations. Rather, they are model input parameters that characterize the entire conformation of the mechanism. To identify motion patterns, we first created event windows of ∼0.5 s in duration surrounding peaks of mandibular depression (i.e., gape), opercular flaring out, or both. We then manually classified the motion within each event window into five types based on the relative amplitudes and timing of peaks: RC wave, caudorostral wave, compressive wave, slow-open wave, and unknown. The traces for each event type were then aggregated by individual and aligned relative to either peak gape (RC and slow-open waves) or peak flaring out (caudorostral and compressive waves). The number of events classified as “unknown” for each individual was: 1 of 100 events (Indiv1), 8 of 103 events (Indiv2), and 11 of 76 events (Indiv3).

To determine whether certain intracranial motion patterns were associated with consistent prey motions, we also measured prey velocity along a RC axis for all feeding trials with marked prey (prey was not marked for 3 of 42 trials). Prey velocities were not included if the prey was sitting on the bottom of the tank or if the prey had entered the esophagus. Prey items were not neutrally buoyant and therefore cannot be used to directly measure fluid flows. However, as long as prey items are suspended in fluid they can provide a somewhat reliable indicator of the direction of fluid flow. Aggregated traces for the slow-open wave and unknown events are not included with the main results because of a lack of data on prey velocities during these events.

## Results

### Anatomical DoFs

To identify the mechanism underlying the function of the mandibular arch, hyoid arch, and shoulder linkage in the channel catfish (Fig. 1C), we first simplified these elements into a mechanism (Fig. 1E) using joint model fitting to determine the type, center, and axes of each joint (Olsen 2019). The resulting mechanism, constructed using four joint types (Fig. 1D) has 17 links, 21 joints, and five loops. According to the Chebychev–Grübler–Kutzbach criterion, a total of 19 DoFs are required to fully specify the conformation of the mechanism (Olsen 2019). For our parameterization of this mechanism, these 19 DoFs represented the following motions (Fig. 2A): five for rotations of links with S-joints at each end about an axis drawn between these joints (arrows labeled O–S in Fig. 2A), three for length changes of the interoperculomandibular ligaments (A and L) and sternohyoideus muscle (B), four for asymmetric motions at the midline (C–F), four for rotations of the left and right opercula (G, H, M, and N), one for mandibular depression (I), one for suspensorial abduction (J), and one for hyoid depression (K).

### DoFs used in motion

We next determined along which of these 19 DoFs there was substantial motion by sequentially freezing each DoF in the mechanism and fitting the resulting model to 3D *in vivo* motion (Fig. 2B). In this way, we quantified fit error as a function of DoFs (Olsen et al. 2017; Stowers et al. 2017). We found that fit error increased non-linearly with decreasing mobility, indicating that DoFs do not have equal effects on model fit. Imposing a constant-length interoperculomandibular ligament (freezing DoF A, Fig. 2A) had no discernible effect on error. Freezing sternohyoideus length changes (DoF B, Fig. 2A) had the next smallest effect, increasing error by 4% on average between the highest- and lowest-parameter models (right axis, Fig. 2A). Freezing the four DoFs that represent midline asymmetric motions (DoFs C–F, Fig. 2A) accounted for an additional 5% of the total difference in error. The two rotations of the left operculum (DoFs G and H, Fig. 2A) increased model error by an additional 10%. Mandibular depression, suspensorial abduction, and hyoid depression (DoFs I–K, Fig. 2A) had the greatest effects, accounting for 29%, 21%, and 31%, respectively, of the remaining difference in error. Among all models, the median RMS error ranged from 0.2 mm to 1.8 mm, with a maximum of 3.1 mm. Measuring error as the Euclidean distance between model and *in vivo*, median errors ranged from 0.2 mm to 2.0 mm, with a maximum of 12 mm.

Despite having 19 potential DoFs, just seven DoFs were sufficient to explain 91% of the motion of the mandibular arch, hyoid arch, and shoulder of channel catfish, a percentage obtained by taking the mean error of the five-DoF model as a percent difference between the errors of our lowest and highest parameter models (Fig. 2B;  Supplementary Movies S1–S3). These seven DoFs (Supplementary Movie S4) include hyoid depression, suspensorial abduction, and mandibular depression and four for left and right opercular abduction and flaring out (since we measured only left-side opercular motion this model corresponds to the five-DoF model in Fig. 2B, however, assuming a comparable range of *in vivo* motion on the right side this would be equivalent to a seven-DoF model). If we were to apply this mechanism model to different individuals of the same or different species we would want the variance of our results to reflect variance among individuals, not model error. Thus, another criterion is whether a best fitting linkage model has an error less than inter-individual variation (Olsen et al. 2017). The seven-DoF bilateral model also satisfied this criterion as it had an error less than the RMS difference in landmark coordinates between each individual and a Procrustes-consensus shape (Zelditch et al. 2004) of 0.91 mm (1.2% head length). While a total of seven DoFs represents less than half of the potential DoFs, it conforms with the theoretical minimum mobility required to fully control the 3D translation and rotation of an ingested food item, supporting the hypothesis that this linkage functions as a manipulation system.

### Manipulation patterns used during feeding

**Fig. 3:**
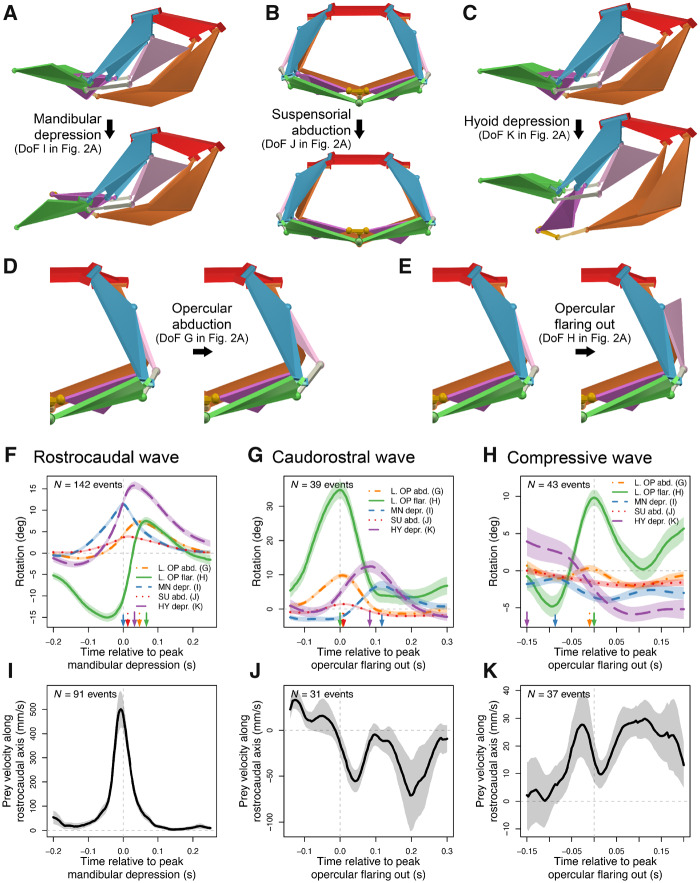
A multi-DoF mechanism enables multiple prey manipulation patterns. Most of the motion in the channel catfish skull can be described by motion along five independent DoFs (Supplementary Movie S4): mandibular depression (**A**), suspensorial abduction (**B**), hyoid depression (**C**), opercular abduction (**D**), and opercular flaring out (**E**). From the motion along these five DoFs, we observed three types of cranial motion patterns: a RC wave (**F**) occurring before or after prey capture, and caudorostral (**G**) and compressive waves (**H**) occurring only after prey capture. Each motion pattern was associated with a different prey velocity profile (**I***–***K**) measured along the RC axis (positive corresponds to caudally directed velocity vector). A RC wave (F) of expansion was associated with a sharp positive peak in prey velocity (I), a caudorostral wave (G) with moderate negative prey velocities (J), and a compressive wave (H) associated with moderate positive prey velocities (K). In (F–K), events from all individuals (*N *=* *3) were pooled with lines and shading indicating mean and standard error, respectively; letters in parentheses correspond to labels in Fig. 2A. Some motion events lacked associated prey velocity data. Vertical arrows along *x*-axis represent the sequence of peaks for each DoF. *Y*-values in (F–H) are on a consistent scale (e.g., peak left opercular flaring out is greater for the caudorostral wave than for the RC wave). All five of the most substantial DoFs were rotations (versus translation) and thus cranial motions are represented in (F–H) solely by rotations. Figures with results for each individual separately are provided in Supplementary Figs. S1–S3.

To evaluate whether motions of this linkage correspond with prey motions, we divided and categorized all *in vivo* motion sequences into different types and superimposed all motions of the same type with their corresponding prey velocity sequence. We identified three consistent motion patterns (Fig. 3): a RC wave (also called an anterior-to-posterior or AP wave; Fig. 3F), a caudorostral wave (Fig. 3G), and a compressive wave (Fig. 3H). The RC wave was characterized by successive peaks of mouth and throat opening from rostral to caudal and a caudal acceleration of the prey item, which peaked in velocity within 10 ms of maximum mandibular depression (Fig. 3I). The caudorostral wave exhibited successive peaks of mouth and throat opening in the opposite order, associated with acceleration of prey rostrally, peaking in velocity 20–200 ms after peak opercular flaring out (Fig. 3J). The compressive wave was characterized by hyoid elevation followed by opercular flaring out and was generally associated with RC acceleration of prey, at velocities an order of magnitude slower than those during a RC wave (Fig. 3K). These waves did not occur in any consistent order across feeding trials, however, there was some consistency in when each wave was used during the feeding: the RC wave was used during and after prey capture, the caudorostral and compressive waves were used only after prey capture, and the caudorostral wave was used both before and after prey were swallowed.

## Discussion

Previous research on suction feeding in fishes has focused primarily on how fish explosively expand their mouth and throat to capture prey during suction feeding (Gibb and Ferry-Graham 2005; Camp et al. 2015). For high-power suction feeders, in particular, so much power is required that the body muscles supply up to 95% of the power by actuating the cranial linkage through attachments on the neurocranium and shoulder (Carroll et al. 2004; Camp et al. 2015). This has led to the useful analogy of a fish skull as an umbrella: capable of expansion and compression but actuated by minimal DoFs (Camp et al. 2015). This is not unreasonable given that a two-DoF robotic model of largemouth bass reproduces *in vivo* motions of the mandible, suspensorium, and hyoid during the opening phase of suction feeding (Kenaley and Lauder 2016) and even a simple one-DoF syringe can both suck in and expel fluid. At the same time, fish have over two dozen smaller cranial muscles that are active and have recognized roles during feeding (Wainwright et al. 1989; Alfaro et al. 2001), implying a higher DoF system.

Our results reconcile these previously competing estimates of mobility in the fish skull by showing that the channel catfish functions as a five-loop, 14-bar prey manipulation mechanism with 19 potential DoFs and substantial motion along at least seven of these DoFs during feeding. Assuming that for every DoF in a motor system under active control there must be at least two muscles (an agonist-antagonist pair), the over 14 cranial muscles associated with the mandibular arch, hyoid arch, and shoulder in fishes bilaterally (Datovo and Bockmann 2010) is in line with a mechanism of at least seven DoFs. Although it was previously known that fishes could generate bidirectional flows (Ferry-Graham 1999; Callan and Sanderson 2003; Van Wassenbergh et al. 2016), our results provide the first quantitative description of the kinematics and mechanism underlying this behavior. We observed channel catfish frequently employ caudorostral flows during feeding to reposition already captured prey within the mouth and some instances to spit out captured prey items. And our finding that this mechanism generates multiple motion patterns indicates that the RC wave of expansion during suction feeding is not simply the result of a passive delay built into the system (Bishop et al. 2008) but rather the result of active control. This suggests that while the body muscles provide most of the power required for suction feeding, the cranial muscles function to locally control when this power is deployed. If the fish skull had fewer DoFs then the relative timing and magnitudes of expansion in different parts of the skull could not be varied to generate the different motion patterns we observed.

Although we tracked the motion of all the skeletal elements that comprise the mandibular arch, hyoid arch, and shoulder, our conclusions should be considered a minimum mobility of the channel catfish skull as there are additional mobile cranial elements not included in this study. For example, fish use the locomotor system to position and orient the head directly within striking distance of prey prior to capture (Holzman et al. 2007; Jacobs and Holzman 2018). In addition, we do not know how much the branchial apparatus (i.e., the gill skeleton) and the pharyngeal jaws contribute to prey manipulation during feeding in channel catfish. The greater variability in prey motions associated with the caudorostral and compressive waves, which occurred only after prey were captured, could be due in part to direct manipulation by the pharyngeal jaws (Wainwright et al. 2012) and changes in the width of the gap between adjacent gill bars, which can regulate flow between the oral and opercular cavities (Lauder 1983). Even for the cranial elements included in this study there were motions that we were unable to measure due to limitations in our motion capture dataset (the eight DoFs frozen *a priori*, see “Materials and Methods” section). While most of these motions are likely to be minor and not expected to change the conclusions of this study, a potential exception is asymmetric opercular motions. Since we were unable to measure motions of both the left and right opercula, it is possible that channel catfish can move their opercula independently or asynchronously as an additional axis of mobility to control the flow of fluid.

The basic mechanism we have shown here is likely generalizable to the vast majority of fishes because these skeletal and ligamentous couplings are ancestral to all of Teleostei, which comprises 96% of the over 30,000 extant fish species (Lauder 1982). Thus, any variation in cranial mobility among teleost fishes is likely to be found elsewhere in the skull. For example, whereas the premaxillae are fused to the neurocranium in channel catfish, several fish lineages have evolved the ability to protrude the premaxillae to extend the oral jaws toward prey items independent of body motions (Staab et al. 2012). In some lineages (e.g., percomorphs), protrusion would presumably not increase mobility as it is assumed to be coupled with mandibular depression (Westneat 1990) while in other lineages (e.g., cypriniforms, some coral reef fishes) protrusion has been shown to be independent of mandibular depression (Konow et al. 2008; Gidmark et al. 2012), increasing system mobility by one DoF. Thus, variation in cranial DoFs across fishes is less likely to occur as modifications within this mechanism than as elaborations upon this mechanism for particular specialized behaviors.

It may, in fact, be surprising that motion of the fish skull can be characterized by so few DoFs given the number of mobile elements. After all, motion of the human arm (excluding the hand), which has many fewer elements, can also be characterized by seven DoFs. However, a key difference between these two systems is that elements in the fish skull are highly interconnected; we identified at least five joint loops in the channel catfish skull (Fig. 1F). It is this high degree of interconnectedness (in addition to the individual joint mobilities) that decreases the mobility of the fish skull from what we might expect given only the number of moving parts (Olsen 2019). The net result is that the motion (and potentially the control) of the fish skull, with 14 mobile elements, is no more complicated than that of a human arm reaching to pick up an object.

The finding that fish skulls possess as much, or more, mobility than the feeding system of tetrapods is consistent with a general and unifying principle that a motor system must have, at a minimum, a mobility that is consistent with the tasks that system performs. This principle unifies biological manipulation systems having radically different designs and operating in different media. For example, the fish skull, primate arm (Alexander 1992), and the primate feeding system (i.e., mandible and tongue; Beautemps et al. 2001; Iriarte-Díaz et al. 2017) all have at least seven DoFs. This principle also provides a different perspective on the evolution of biomechanical systems. For example, in moving from water to land tetrapods transitioned from hydraulic intraoral transport to the use of a flexible tongue (Reilly and Lauder 1990; Heiss et al. 2018). From the perspective of minimum motor system mobility, the evolution of a flexible tongue of tetrapods was both an adaptation for directly manipulating prey outside of a fluid medium and a means of maintaining the total mobility of the feeding apparatus in compensation for the loss of effective fluid-manipulation by a kinetic skull in air.

To our knowledge, this five-loop mechanism in the channel catfish is unlike any current engineered mechanisms, not just in its configuration and constituent joint types but also in combining high mobility with flow-based manipulation. Existing flow-based manipulation systems, such as those that translate suspended particles, have a maximum of two DoFs, and are capable of controlling only two axes of translation (Tanyeri and Schroeder 2013). The only manipulation systems with mobility comparable to the fish skull are systems that rely on direct, physical contact, such as four-DoF parallel manipulators (Wu and Yin 2008), seven-DoF robotic arms (Koch et al. 2018), or a five-DoF gripper that combines four DoFs of direct contact through robotic fingers with one-DoF suction flow (Stuart et al. 2014). Such has been the difficulty in designing an underwater flow-based manipulation system that a frequent solution is to simply employ the mainstay of air-based manipulation, the robotic arm, underwater (Koch et al. 2018). Engineering a manipulation system based on the fish head may allow more precise control than current flow-based systems, given at least seven DoFs, without the need to make direct contact with an object (i.e., control entirely through fluid flow manipulation). The mechanism proposed here, evolved precisely for such tasks, provides a template for a new type of bio-inspired, flow-based manipulation system with sufficient mobility to perform complicated underwater manipulation tasks.

## Supplementary Material

obaa031_Supplementary_Data
