# Supplementary material for: Multiple Degrees of Freedom in the Fish Skull and Their Relation to Hydraulic Transport of Prey in Channel Catfish
Source: Integr Org Biol. 2020 Nov 10;2(1):obaa031. doi: 10.1093/iob/obaa031 (PMC7671092; doi:10.1093/iob/obaa031)
Supplement: obaa031_Supplementary_Data [file obaa031_supplementary_data.zip › Supplement.pdf]

Supplemental figures and explanation of supplemental data file for

The channel catfish head functions as a five-loop, 14-bar prey manipulation mechanism during feeding

Aaron M. Olsen, L. Patricia Hernandez, Elizabeth L. Brainerd

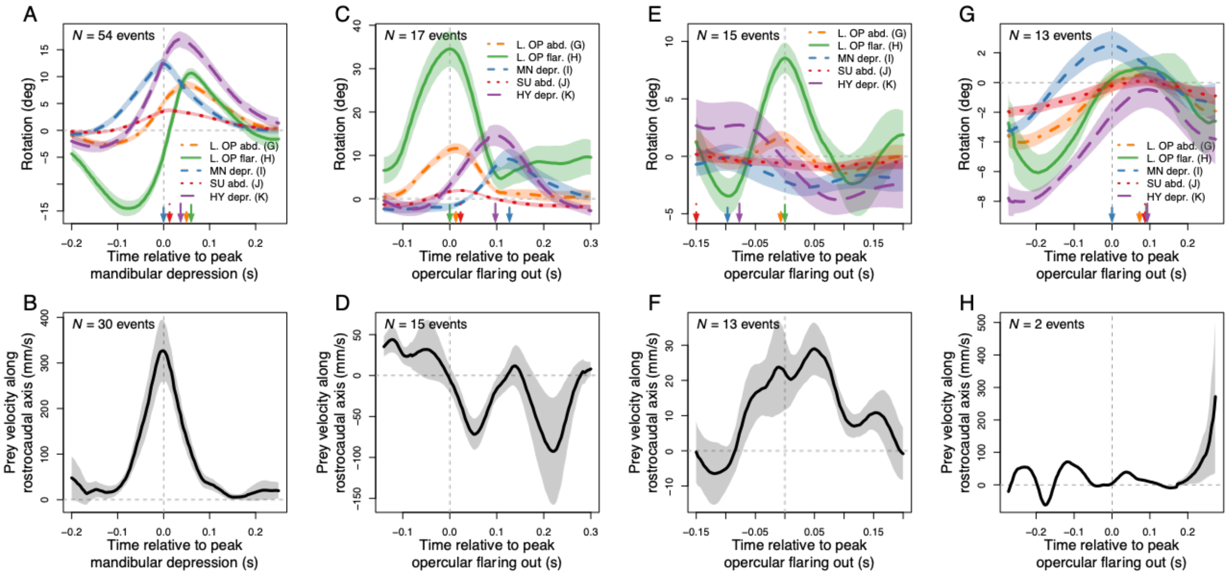

Fig. S1. Figure equivalent to Fig. 3 in the main text but only showing data from Indiv1. We observed three types of consistent cranial motion patterns (*A*,*C*,*E*), characterized by the five most substantial DoFs (letters in parentheses correspond to labels in Fig. 2). Each motion pattern was associated with a different prey velocity profile (*B*,*D*,*F*), measured only along the rostrocaudal axis (positive corresponds to caudally directed velocity vector). A rostrocaudal wave (*A*) wave of expansion was associated with a sharp positive peak in prey velocity (*B*), a caudorostral wave (*C*) with moderate negative prey velocities (*D*), and a compressive wave (*E*) associated with moderate positive prey velocities (*F*). A fourth type of motion pattern (*G*) was also observed, similar to the rostrocaudal wave but having slower and lower magnitude motions; we call this a 'slow open'. Slow open behaviors were performed while the individual was seeking the prey (before making contact with the prey). Therefore most of these motions could not be associated with any prey motion and number of associated prey motions (*H*) are too few to draw conclusions about the effect of this behavior on fluid flows. Lines and shading indicating mean and standard error, respectively. Some motion events lacked associated prey velocity data. Vertical arrows along x-axis represent the sequence of peaks for each DoF. Y-values are on a consistent scale across *A*, *C*, *E*, and *G* (e.g. peak left opercular flaring out is greater for the caudorostral wave than for the rostrocaudal wave).

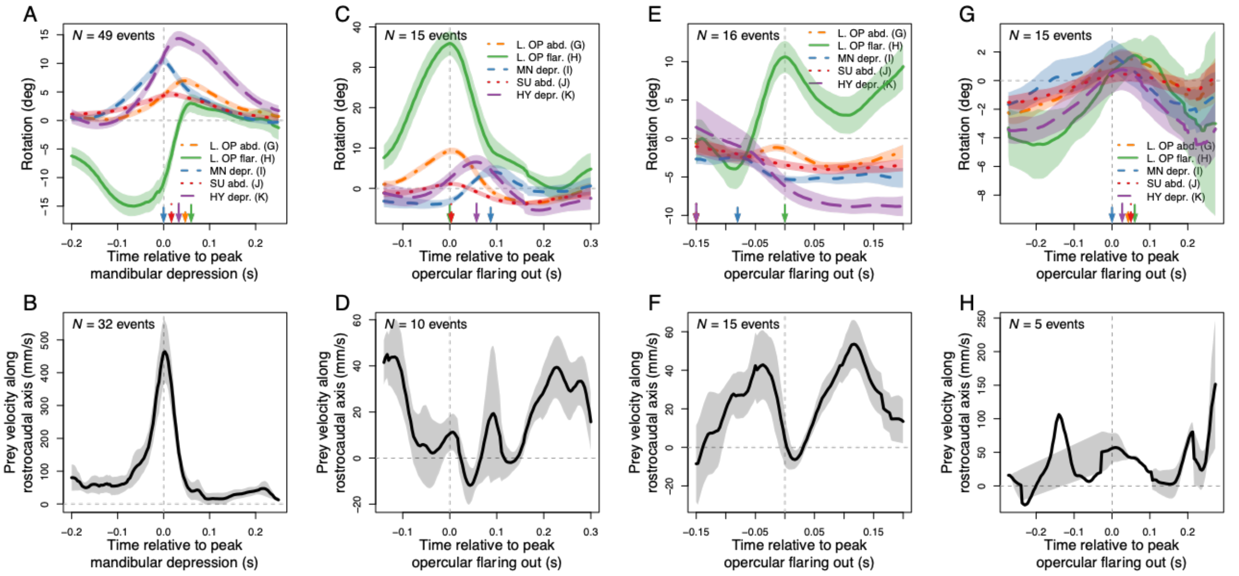

Fig. S2. Figure equivalent to Fig. 3 in the main text but only showing data from Indiv2. We observed three types of consistent cranial motion patterns (*A*,*C*,*E*), characterized by the five most substantial DoFs (letters in parentheses correspond to labels in Fig. 2). Each motion pattern was associated with a different prey velocity profile (*B*,*D*,*F*), measured only along the rostrocaudal axis (positive corresponds to caudally directed velocity vector). A rostrocaudal wave (*A*) wave of expansion was associated with a sharp positive peak in prey velocity (*B*), a caudorostral wave (*C*) with moderate negative prey velocities (*D*), and a compressive wave (*E*) associated with moderate positive prey velocities (*F*). A fourth type of motion pattern (*G*) was also observed, similar to the rostrocaudal wave but having slower and lower magnitude motions; we call this a 'slow open'. Slow open behaviors were performed while the individual was seeking the prey (before making contact with the prey). Therefore most of these motions could not be associated with any prey motion and number of associated prey motions (*H*) are too few to draw conclusions about the effect of this behavior on fluid flows. Lines and shading indicating mean and standard error, respectively. Some motion events lacked associated prey velocity data. Vertical arrows along x-axis represent the sequence of peaks for each DoF. Y-values are on a consistent scale across *A*, *C*, *E*, and *G* (e.g. peak left opercular flaring out is greater for the caudorostral wave than for the rostrocaudal wave).

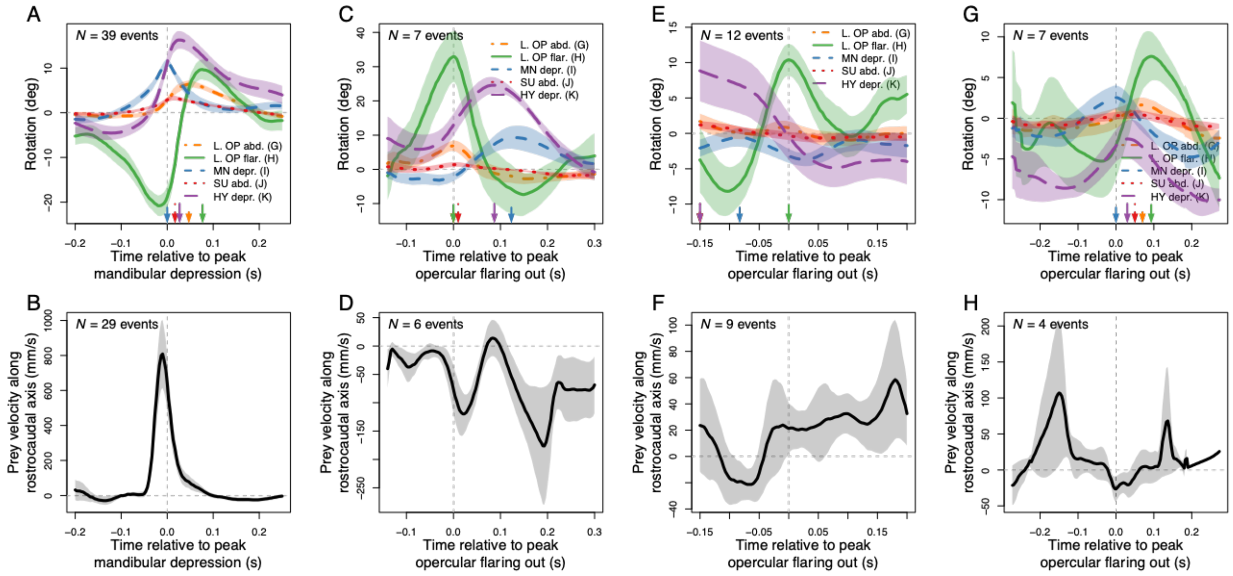

Fig. S3. Figure equivalent to Fig. 3 in the main text but only showing data from Indiv3. We observed three types of consistent cranial motion patterns (*A*,*C*,*E*), characterized by the five most substantial DoFs (letters in parentheses correspond to labels in Fig. 2). Each motion pattern was associated with a different prey velocity profile (*B*,*D*,*F*), measured only along the rostrocaudal axis (positive corresponds to caudally directed velocity vector). A rostrocaudal wave (*A*) wave of expansion was associated with a sharp positive peak in prey velocity (*B*), a caudorostral wave (*C*) with moderate negative prey velocities (*D*), and a compressive wave (*E*) associated with moderate positive prey velocities (*F*). A fourth type of motion pattern (*G*) was also observed, similar to the rostrocaudal wave but having slower and lower magnitude motions; we call this a ‘slow open’. Slow open behaviors were performed while the individual was seeking the prey (before making contact with the prey). Therefore most of these motions could not be associated with any prey motion and number of associated prey motions (*H*) are too few to draw conclusions about the effect of this behavior on fluid flows. Lines and shading indicating mean and standard error, respectively. Some motion events lacked associated prey velocity data. Vertical arrows along x-axis represent the sequence of peaks for each DoF. Y-values are on a consistent scale across *A*, *C*, *E*, and *G* (e.g. peak left opercular flaring out is greater for the caudorostral wave than for the rostrocaudal wave).

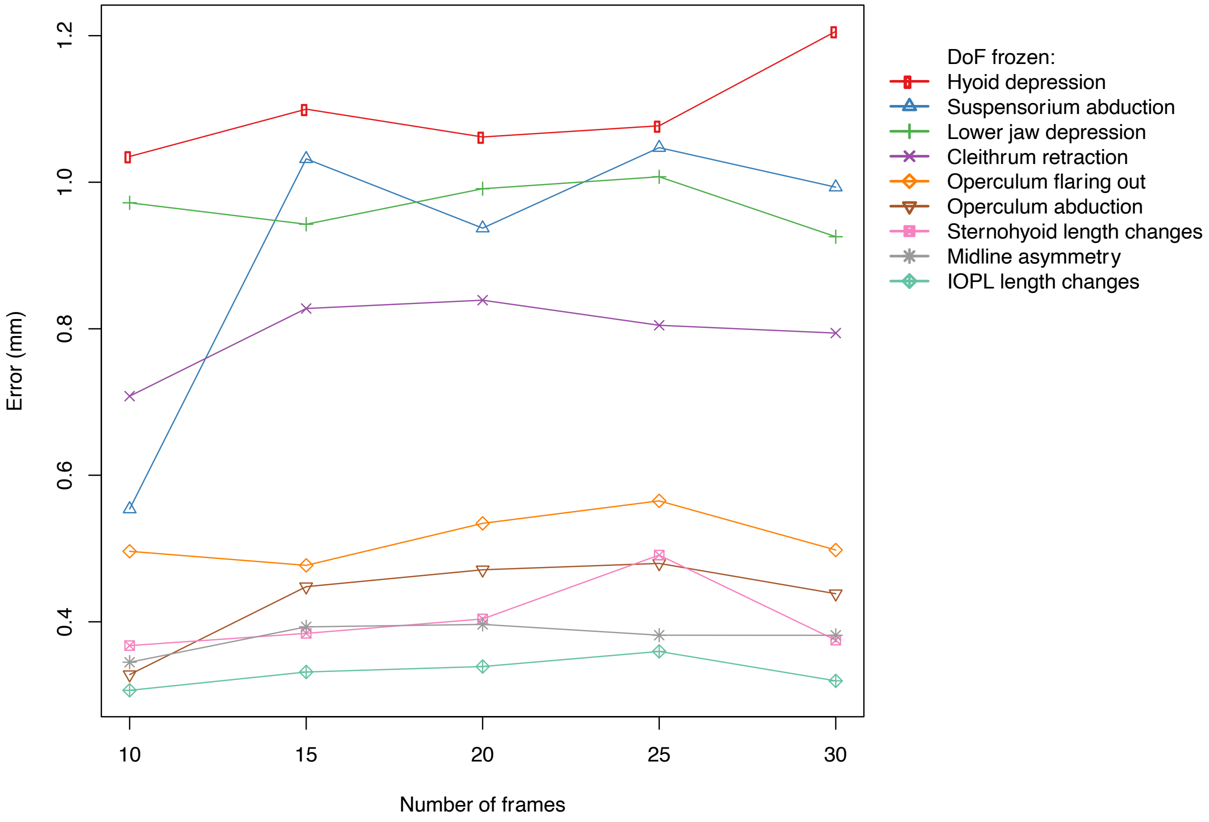

Fig. S4. Mechanism model fit error versus number of frames for Indiv1. Because our motion dataset included thousands of frames and because the optimization steps of our model fitting are computationally intensive we needed to choose a subset of frames from our whole motion for model fitting. By examining how model fit errors varied across the number of frames chosen for the fitting we determined a sufficient number of frames to include in the fitting. We used principal components analysis to create a lower dimensional conformation space from the motion data and then chose maximally dispersed points (each of which represents a different frame from the motion dataset) within that conformation space as the frame subset. Because we filmed each individual perform multiple feeding events we expect substantial replication within the motion data. Thus, as long as the subset of frames is chosen in such a way that whole conformation space of the skull is evenly sampled, a reduced number of frames should still be representative of the conformation space of the entire motion dataset. This figure shows the fit errror (y-axis) of nine different models (represnted by different colors and symbols) using 10, 15, 20, 25, and 30 frames sampled in a maximally dispersed manner from the total motion dataset. Each model is the full-DoF model but with one DoF (or category of DoFs) frozen; for example, the line labeled "Hyoid depression" corresponds to the full DoF model but with hyoid depression disallowed (frozen). Note that DoFs that have a greater effect on model fit generally have larger errors across all numbers of frames. Because there appeared to be no consistent trend in error with the inclusion of more than 15 frames we determined that 15 frames was sufficiently representative for all model fitting.

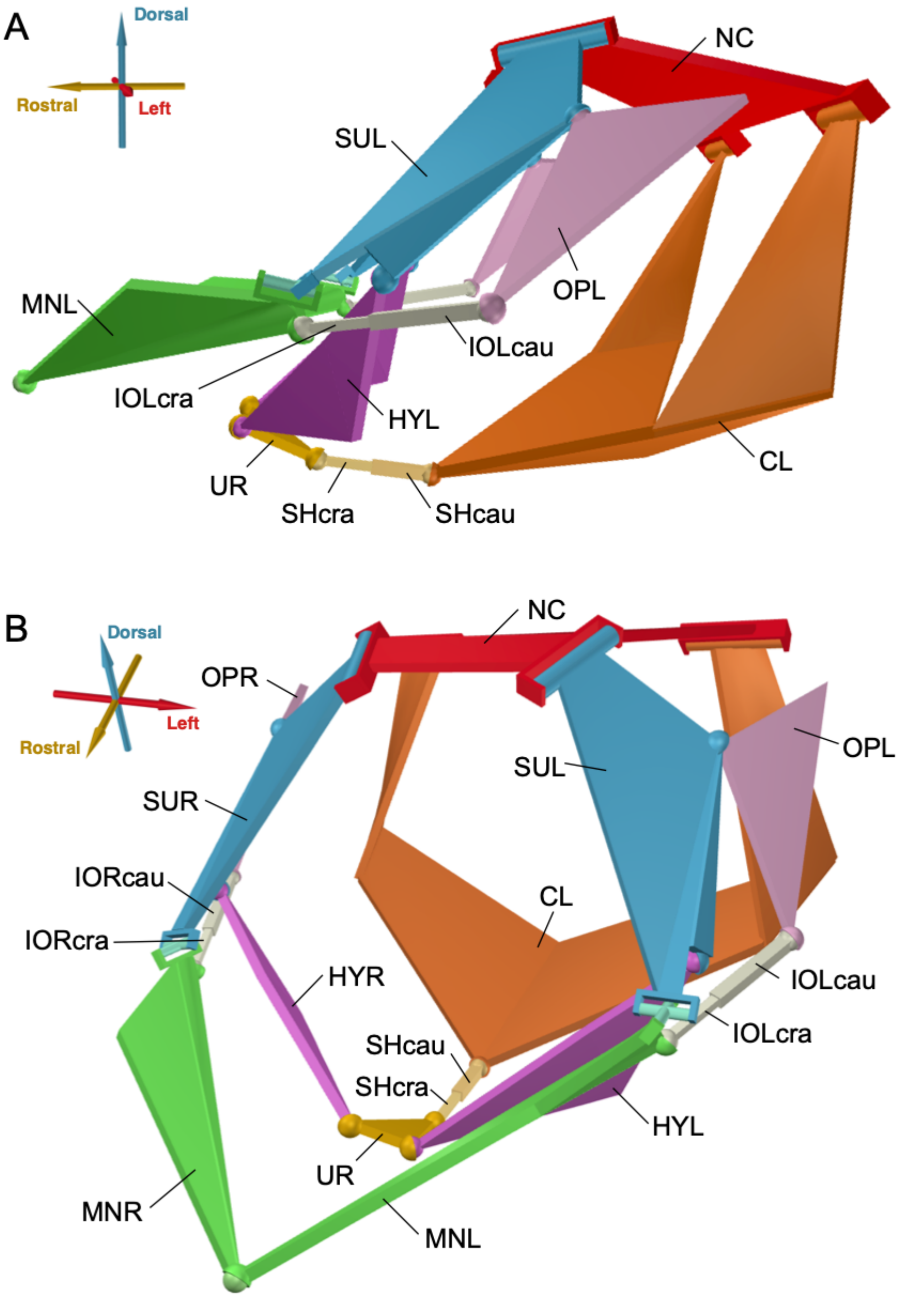

Fig. S5. Mechanism model with labeled links. This figure provides a reference for the model parameters listed in Tables S1-3.

Note for Tables S1-3. The following three tables provide parameters for the mechanism model for each individual. The **Joint Centers** are XYZ-coordinates; for rotational joints these coordinates represent the center of rotation for that joint whereas for the prismatic joints the coordinates are simply the midpoint between the joints on each side of the prismatic joint. The two links joined by each joint are indicated by an abbreviation (see Fig. S5 for a link abbreviation key) separated by an underscore. For example, the 'NC_SUL' joint is the joint between the neurocranium (NC) and the left suspensorium (SUL). The **Joint Axes** are normal vectors indicating the orientation of the joint axes for each rotational joint. If the joint type is a revolute type joint (a hinge) then only a single axis is given (aor1). If the joint is a universal type joint (a saddle joint) then two axes are given (aor1 and aor2), with aor1 being the axis about which there is a greater magnitude of rotation. If the joint is a spherical type joint (a ball-and-socket joint) then no axes are given since the links can rotate about any axis. The last section, **General Parameters**, provides the XYZ coordinate of the neurocranium centroid and the three body axes (as unit vectors) determined from the shape of the neurocranium. Model parameters are bilateral for Indiv1 (solely to create a bilateral visualization) and mostly unilateral for Indiv2 and Indiv3.

Table S1. Mechanism model parameters for Indiv1. See **Note for Tables S1-3** on previous page.

	X	Y	Z
**Joint Centers**			
IOLcau IOLcra	-9.903	-61.369	-88.771
IORcau IORcra	-64.136	-64.692	-86.883
MNL IOLcra	-12.848	-63.288	-81.558
MNL MNR	-35.386	-60.801	-50.057
MNR IORcra	-60.565	-66.204	-79.684
NC CL	-19.593	-40.005	-124.719
NC SUL	-26.639	-35.983	-99.750
NC SUR	-50.097	-38.347	-99.769
OPL IOLcau	-6.957	-59.450	-95.983
OPR IORcau	-67.708	-63.181	-94.082
SH UR	-37.082	-62.001	-73.400
SHcau SHcra	-35.050	-63.913	-77.694
SHcra UR	-35.265	-61.818	-73.399
SUL HYL	-12.534	-58.681	-88.643
SUL MNL	-12.632	-60.220	-82.334
SUL OPL	-14.679	-44.088	-102.202
SUR HYR	-62.054	-60.571	-87.401
SUR MNR	-61.695	-63.258	-80.010
SUR OPR	-61.965	-47.267	-101.015
UR HYL	-33.197	-58.945	-66.150
UR HYR	-39.913	-58.638	-65.973
**Joint Axes**			
NC CL aor1	0.992	0.104	-0.070
NC SUL aor1	-0.309	-0.274	0.911
NC SUR aor1	0.356	-0.207	0.911
SUL MNL aor1	0.986	-0.079	-0.149
SUL MNL aor2	0.168	0.523	0.836
SUR MNR aor1	-0.938	-0.339	-0.074
SUR MNR aor2	-0.202	0.360	0.911
**General Parameters**			
NC midline pt	-37.704	-43.887	-83.028
NC dorsoventral axis	-0.119	0.974	0.195
NC rostrocadual axis	-0.027	-0.199	0.980
NC transverse axis	0.992	0.112	0.050

Table S2. Mechanism model parameters for Indiv2. See **Note for Tables S1-3** on page prior to **Table S1**.

	X	Y	Z
**Joint Centers**			
IOLcau IOLcra	-16.307	-63.826	-54.396
MNL IOLcra	-19.424	-65.094	-47.612
MNL MNR	-42.147	-59.694	-18.282
NC CL	-25.710	-43.494	-92.656
NC SUL	-38.931	-45.197	-47.089
OPL IOLcau	-13.191	-62.557	-61.179
SH UR	-40.862	-63.015	-40.862
SHcau SHcra	-42.069	-64.325	-45.188
SHcra UR	-42.079	-63.011	-40.866
SUL HYL	-16.456	-59.655	-56.621
SUL MNL	-19.993	-61.664	-47.988
SUL OPL	-17.821	-48.754	-65.474
UR HYL	-40.309	-59.625	-33.527
**Joint Axes**			
NC CL aor1	0.999	-0.050	0.006
NC SUL aor1	0.343	0.309	-0.887
SUL MNL aor1	0.993	-0.075	-0.086
SUL MNL aor2	0.114	0.584	0.804
**General Parameters**			
NC midline pt	-41.747	-44.120	-55.255
NC dorsoventral axis	0.017	0.995	0.093
NC rostrocadual axis	-0.014	-0.093	0.996
NC transverse axis	1.000	-0.018	0.013

Table S3. Mechanism model parameters for Indiv3. See **Note for Tables S1-3** on page prior to **Table S1**.

	X	Y	Z
**Joint Centers**			
IOLcau_IOLcra	-50.203	-89.456	-73.679
MNL_IOLcra	-54.828	-92.118	-64.636
MNL_MNR	-79.538	-90.800	-31.224
NC_CL	-61.085	-67.978	-117.114
NC_SUL	-74.664	-67.646	-64.522
OPL_IOLcau	-45.579	-86.794	-82.722
SH_UR	-79.390	-92.708	-57.173
SHcau_SHcra	-77.979	-93.520	-61.337
SHcra_UR	-78.244	-92.606	-57.123
SUL_HYL	-52.745	-87.476	-76.109
SUL_MNL	-50.634	-87.378	-66.043
SUL_OPL	-51.857	-70.076	-89.429
UR_HYL	-76.618	-86.237	-48.325
**Joint Axes**			
NC_CL_aor1	0.995	0.087	-0.045
NC_SUL_aor1	-0.305	-0.269	0.913
SUL_MNL_aor1	-0.982	-0.012	-0.190
SUL_MNL_aor2	0.162	-0.580	-0.799
**General Parameters**			
NC_midline_pt	-80.659	-68.778	-71.498
NC_dorsoventral_axis	-0.034	0.983	0.182
NC_rostrocadual_axis	0.060	-0.180	0.982
NC_transverse_axis	0.998	0.044	-0.053

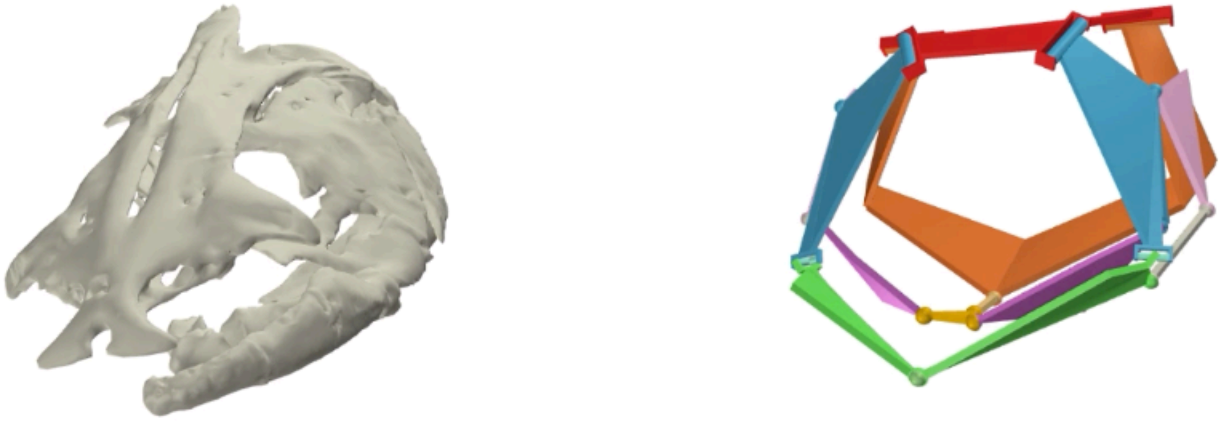

Movie S1. *In vivo* motion versus best-fit mechanism model side by side. This movie shows an animation of *in vivo* motion from a single trial (left) and the mechanism model fit to those *in vivo* data animated using seven DoFs (right). We only collected *in vivo* motion data from the right side of the skull but because three of these structures extend to the midline we were able to reconstruct motion of the mandible and suspensorium on the right side. Animation of the right operculum in the mechanism model is not based on *in vivo* motion and is simply a mirror of the left side for visual completeness; we were not able to assess whether opercular motions are bilaterally symmetric. Without motions of the right operculum (two DoFs) the mechanism is driven by the five principal DoFs discussed in the paper (see Fig. 3).

Access here: https://drive.google.com/file/d/1GyBn9H-oVvBGe_Umy7Al0LMJ5XQ7fzTi/view

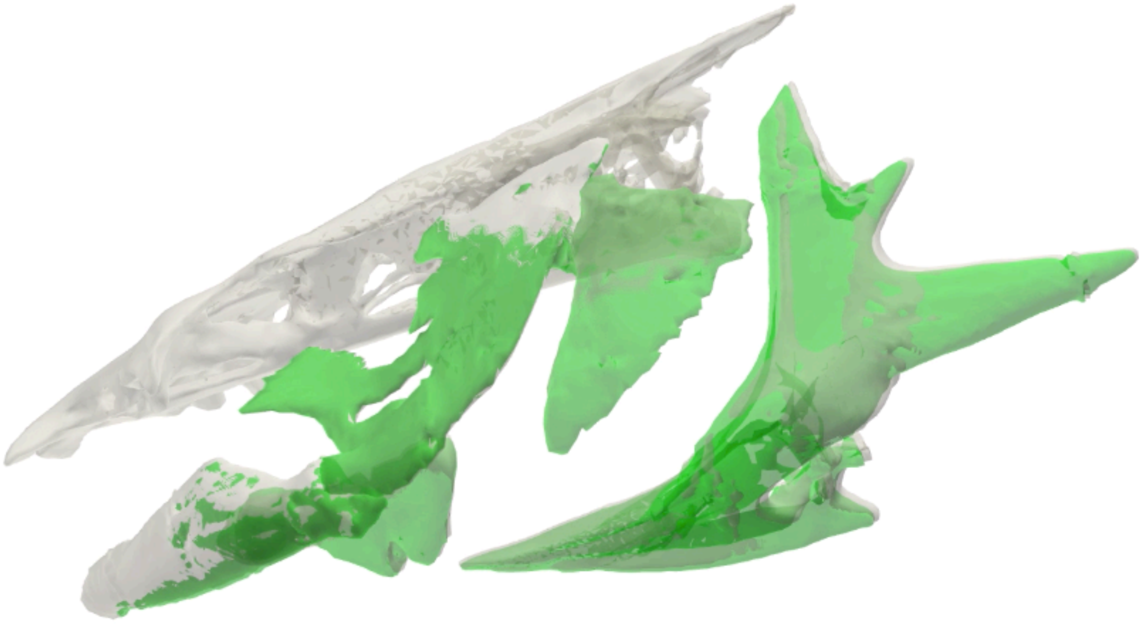

Movie S2. *In vivo* motion versus best-fit mechanism model superimposed (lateral view). This movie shows *in vivo* motion for a single trial (in white) and motion from a five-DoF mechanism model superimposed onto this motion (in green) from a lateral view. The broad overlap between the *in vivo* and model motion shows that five DoFs are sufficient to capture most of the *in vivo* motion. The five DoFs used to drive the mechanism are those highlighted in Fig. 3.

Access here: https://drive.google.com/file/d/1b4TzjZE6Jriknj2cTfFg7StJhBNW9H8r/view

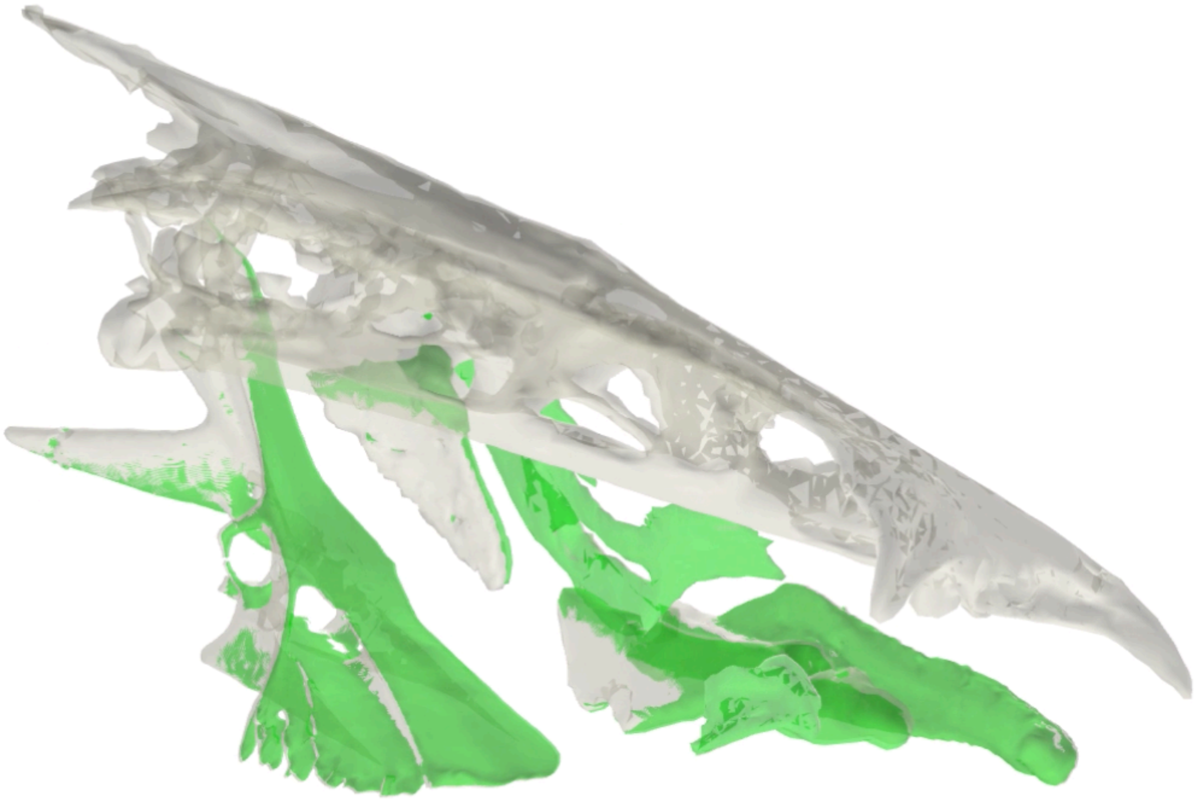

Movie S3. *In vivo* motion versus best-fit mechanism model superimposed (medial view). This movie is identical to Movie S2 except from a medial perspective. This movie shows *in vivo* motion for a single trial (in white) and motion from a five-DoF mechanism model superimposed onto this motion (in green) from a medial view. The broad overlap between the *in vivo* and model motion shows that five DoFs are sufficient to capture most of the *in vivo* motion. The five DoFs used to drive the mechanism are those highlighted in Fig. 3.

Access here: https://drive.google.com/file/d/1g4pu62HC_i3hnMXkPr5wDVIzWGWQnmTP/view

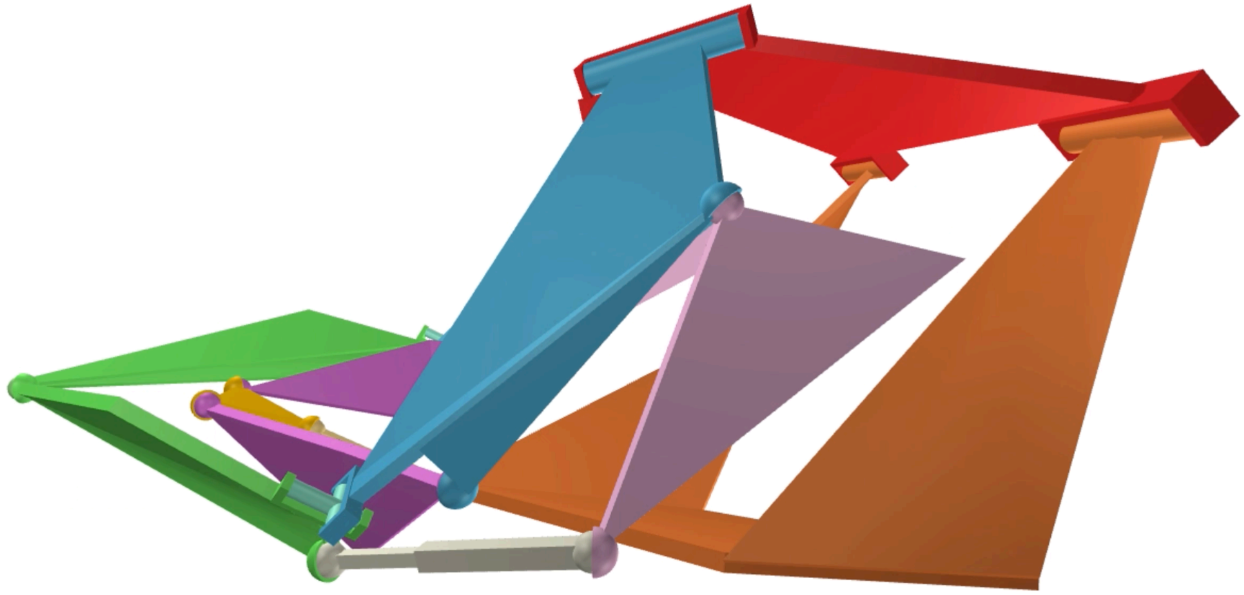

Movie S4. Mechanism DoFs with the greatest effect on model fit. This movie shows the five DoFs of the mechanism included in Fig. 3 animated separately. These five DoFs are animated in the movie in the following order: (1) mandibular depression coupled to operculum retraction, (2) suspensorium abduction, (3) hyoid depression coupled to cleithrum retraction, (4) operculum abduction, (5) operculum flaring out.

Access here: https://drive.google.com/file/d/1NpF8ZWRe3y5xmYGtsjaPl6Lqa9ASE7BP/view

Fig 3 data.csv

Access here: https://drive.google.com/open?id=1NykAeC8GqZc43-CHcoil5Rf4dadatx5-

The supplemental file named ‘Fig 3 data.csv’ contains all of the motion date shown in Fig. 3. The motion data in this file are the motions output from fitting the mechanism to *in vivo* motion, using the five DoFs included in Fig. 3. The original raw motion data are accessible from xmaportal.org in the study ‘Catfish Suction Feeding’ (ID: BROWN61). The motion data are separated into events based on peak mandibular depression or peak operculum flaring out. The rows in ‘Fig 3 data.csv’ each correspond to a time point in the motion sequence. The columns in ‘Fig 3 data.csv’ and a description of their contents are described below:

**Column name**	**Description**
trial	The trial from which the motion data are taken. These trial names correspond to those used.
time_s	The timestamp for the corresponding row (in seconds).
behavior	The behavior the animal was performing at that time, either capture (prey capture) or transport (moving the prey item through the mouth or throat).
SU_abd	Magnitude of suspensorium abduction (in degrees).
MN_depr	Magnitude of mandibular depression (in degrees).
LOP_flar	Magnitude of left operculum flaring out (in degrees).
HY_depr	Magnitude of hyoid depression (in degrees).
LOP_abd	Magnitude of left operculum abduction (in degrees).
prey_loc	Location of the prey at the corresponding time. Possible values are: ‘tank’ (prey is in the tank and not being moved by the fish), ‘oral’ (prey is actively being sucked into the mouth or is in the mouth but rostral to the approximate rostral-most position of the pharyngeal jaws), ‘pha’ (prey is caudal to the approximate rostral-most position of the pharyngeal jaws but rostral to the approximate position of the esophageal opening), ‘eso’ (the prey has passed caudal to the approximate opening of the esophagus), ‘none’ (the prey item was not marked for that trial so the position of the prey is not known), ‘spit’ (prey item is being spit out of the mouth).
prey_vel_tank_sm	Smoothed velocity of the prey item relative to the tank (in mm/second).
prey_pos_rc_sm	Smoothed position of the prey along the rostrocaudal axis of the neurocranium (in mm/second).

prey_vel_rc_sm	Smoothed velocity of the prey item along the rostrocaudal axis of the neurocranium (in mm/second).
time_s_to_LOP_flar_peak	The time to peak flaring out of the left operculum (in seconds).
time_s_to_MN_depr_peak	The time to peak depression of the mandible (in seconds).
event_num	The event number for the corresponding rows.
individual	The individual from which the data were collected. Possible values are: Indiv1, Indiv2, and Indiv3
event_type	The type of event. Possible values are: ‘RC’ (rostrocaudal wave), ‘CR’ (caudorostral wave), ‘CW’ (compressive wave), ‘SO’ (slow open wave), ‘U’ (unidentified).
